# Effects of Parental Involvement in a Preschool-Based Eye Health Intervention Regarding Children’s Screen Use in China

**DOI:** 10.3390/ijerph182111330

**Published:** 2021-10-28

**Authors:** Shu-Mei Liu, Fong-Ching Chang, Cheng-Yu Chen, Shu-Fang Shih, Bo Meng, Eric Ng, Chia-Hsuan Hsu, Yi-Te Chiang, Xiao-Jie Mao, Ming-Yan Yi, Ben LePage, Wei-Ta Fang

**Affiliations:** 1 Department of Preschool Education, Jing Hengyi College of Education, Hangzhou Normal University, Hangzhou 311121, China; liushumei@hznu.edu.cn; 2 Department of Health Promotion and Health Education, National Taiwan Normal University, Taipei 106, Taiwan; t09004@ntnu.edu.tw; 3 Department of Health Administration, College of Health Professions, Virginia Commonwealth University, Richmond, VA 23298, USA; shihs2@vcu.edu; 4 Department of Surgery, Shanxi Provincial People’s Hospital, Taiyuan 030012, China; mengbo0528@163.com; 5 School of Business, University of Southern Queensland, Toowoomba, QLD 4350, Australia; eric.ng@usq.edu.au; 6 Graduate Institute of Environmental Education, National Taiwan Normal University, Taipei 116, Taiwan; d05625002@ntu.edu.tw (C.-H.H.); faratajiang@gmail.com (Y.-T.C.); benlepage2@gmail.com (B.L.); 7 Health Care Center, The First Kindergarten in Fengtai District, Beijing 100071, China; yiwushimaoxiaojie@126.com (X.-J.M.); ymyanlsh_zhu@126.com (M.-Y.Y.); 8 Academy of Natural Sciences, Philadelphia, PA 19103, USA

**Keywords:** eye health, screen use, children, preschool, parent involvement

## Abstract

In this digital era, young children spend a considerable amount of time looking at telephone, tablet, computer and television screens. However, preventative eye health behavior education could help avoid and relieve asthenopia. The effects of parental influence on their children’s eye health behavior through the preschool eye health education intervention program were examined. The Health Belief Model was used to develop parental involvement strategy and eye health curriculum. The study was conducted in a large public preschool with five branches in Beijing, China. A total of 248 parent–child pairs participated in the baseline and follow-up surveys, of which 129 were in the intervention group and 119 were in the comparison group. The generalized estimating equation analysis results indicated that parental involvement in preschool-based eye health intervention on screen uses had positive influence on parents’ eye health knowledge, cues to action, and parenting efficacy. The intervention program also had positive effects on the increasing level of children’s eye health knowledge, beliefs, cues to action, self-efficacy, and behaviors. The results supported the implementation of a preschool-based eye health intervention program with parental involvement, which could potentially enhance children’s and parents’ eye health beliefs and practices.

## 1. Introduction

Children’s eye health has been found to be strongly associated with the quality of learning and achievement in school, which impacts their quality of life and future economic productivity [1]. Globally, there are an estimated 19 million children with vision impairments, and the majority of these are either preventable or can be alleviated [2]. Children’s eye health is a growing public health problem worldwide, and this is particularly evident in East Asian countries [3]. In 2019, the overall myopia rate of children and adolescents in China was 53.6%, and the prevalence of poor vision in preschoolers was 14.5% [4]. Furthermore, more than one-third of first-grade students in Beijing have poor vision [5]. Previous studies [6,7] suggest that myopia is more likely to progress into more severe myopia in later childhood years, and may result in an irreversible loss of vision [8].

In this digital era, young children spend a considerable amount of time looking at screens (e.g., computers, tablets, smartphones, and televisions) [9,10,11]. Studies conducted in a number of countries indicate that about two-thirds of the children exceeded the one hour per day screen time recommendation proposed by the American Academy of Pediatrics [11,12,13]. Other studies [14,15,16,17,18] also noted that young children who spend extended periods of time looking at screens tend to experience intense asthenopia (eye strain), which has contributed to increased asthenopia in youths [19]. In addition, recent studies indicate that screen use may impact the level of myopia [20,21,22].

However, preventative eye health behaviors could potentially help avoid and relieve asthenopia [23]. Although early eye health education in schools is regarded as an effective method for improving children’s eye health knowledge, attitudes, and behaviors, there is limited evidence of eye health programs being included as part of the educational curriculum design and development [24]. The government of China has acknowledged the importance of eye health education and has responded with a proposed comprehensive plan to help prevent and control myopia in children and adolescents [25].

Parents with higher levels of risk perception and greater parental efficacy are more likely to mediate their child’s eye care behavior [26]. Moreover, children with high academic performance tend to have a higher level of risk perception, and those whose parents provide a higher level of mediation are more likely to engage in better eye care behavior [26]. Studies have also shown that a parent’s low level of health literacy is linked to children’s poor health-related knowledge, behaviors, and outcomes [27,28]. Therefore, there is an increasing need and responsibility for parents to foster their children’s health literacy skills [29]. Parental involvement has been widely acknowledged as a vital strategy for improving children’s development and is particularly effective in early childhood [30]. Although parental involvement has been shown to improve the effects of health behavior intervention [31], there is a lack of research examining parental involvement in children’s health literacy skills.

This present study aims to fill this gap by using the Health Belief Model (HBM) to better understand parental influence on their children’s eye health behavior through the preschool eye health education in China. This study posits that a preschool-based eye health intervention education program that is supported by parental involvement could positively improve children’s and their parents’ eye health knowledge.

## 2. Materials and Methods

### 2.1. Preschool Eye Health Intervention Education Curriculum

This study applied the HBM to parental involvement in a preschool eye health program to develop the children’s eye health curriculum, parent resource materials, and parent–child co-learning materials. The HBM theory is a model that explains and predicts preventive eye health behaviors [32], which includes health knowledge, health beliefs (perceived susceptibility, perceived severity, perceived benefits, perceived barriers), cues to action, self-efficacy and health behaviors, and incorporates the concept of self-efficacy that has been rooted in Bandura’s Social Cognitive Theory [33]. The HBM and self-efficacy theories have been widely used in the field of eye health research [34,35]. Parental involvement includes parenting, volunteering, communication, learning at home, decision making, and community collaboration [36]. Based on previous studies and the plan for comprehensive prevention and control of myopia in children and adolescents proposed by the government of China [25], we have simplified the eye health methods into a “1-2-3 strategy” that is comprised of the following: “Screen time should be less than 1 h per day,” “Outdoor activity should be more than 2 h per day,” and “Each near-work activity should not exceed 30 min”. The eye health intervention education curriculum in the present study consists of four modules, that are delivered through ten half-h lessons and activities.

Module 1: “Knowing how electronic screens affect kids’ eyes” aims to increase parents’ and children’s eye health knowledge, susceptibility, and severity of screen overuse and myopia. The methods used to deliver this module include lectures, games, stories, peer-to-peer sharing, singing, and reading parent–child learning leaflets. Module 2: “Life skills training to protect eyes from electronic screen overuse harm”, which enhances children’s life skills by implementing eye health strategies to reduce electronic screen overuse through storytelling and role-playing. Module 3 activities emphasize the need for “improving eye health self-efficacy” that is focused on enhancing children’s eye health self-efficacy through observation and performance of eye care related songs. Module 4 is related to “joining outdoor activities” where the time spent outside is increased and the benefits of eye health can be gained by engaging in outdoor activities such as ball games and throwing frisbees.

Prior to the commencement of the eye health intervention education curriculum, teachers from the preschools had to attend a one-week training workshop on effective eye health instructions conducted by the researcher. The trained teachers implemented the eye health intervention program with parental involvement between 1 and 30 April 2019. During these intervention classes, teachers had used a variety of techniques and activities such as formal instructions, demonstrations, games, stories, role play, peer sharing, and positive reinforcement to achieve the goals of the Modules.

Furthermore, parental resource booklets and leaflets related to children’s eye health were also distributed. Resource booklets for parents included topics on eye health knowledge, risk factors for myopia, the impact of screen overuse, and parenting strategies to protect children’s eye health. Parenting strategies included being a role model, monitoring children’s near-work activity, supervising their eye health behavior, communicating, and negotiating family rules, and creating a supportive environment for them.

For four weeks, the children were asked to take home eye health leaflets, complete four homework assignments incorporating parent–child activities, and return the parents’ feedback to the teacher the following week. The contents of the four parent–child learning leaflets included the following: (1) making eye health decisions with the child and writing down their name on the eye health declaration leaflets; (2) telling stories that taught ways to preserve eye health; (3) communicating with children and setting family eye health rules; and (4) collaborating with children to list the family rules mentioned on posters at home. In addition, an online resource package that included an eye health knowledge video and an ophthalmologist’s eye health lecture video was provided to the parents.

### 2.2. Design

This study adopted a quasi-experimental design conducted at five Beijing preschools between March and May 2019, in which the principals and teachers had agreed to participate. Parents were invited to participate in the study, given consent forms and informed that the data gathered would be published, but names would remain confidential and anonymous. Ten preschool classes participated and were randomly assigned to the intervention (four classes) or comparison (six classes) group. Pre- and post-surveys of the children and parents were conducted in March 2019 (baseline) and May 2019 (follow-up), respectively, to assess the effects of the intervention program.

The researchers visited the preschools to conduct the surveys. Working with children can always introduce unexpected results and biases to the results. Therefore, surveys with children were conducted through face-to-face group interviews in their schools and the researchers read the questionnaire items to the children. The children were not accompanied by their parents. In contrast, parents were asked to complete the survey through the “Wen Juanxing” online platform via the social app WeChat.

### 2.3. Participants

Two hundred sixty children between 5 and 6 years old, and their parents, were invited to participate in the study. The intervention and comparison groups were represented by 137 and 123 parent–child groups, respectively. While the baseline survey received 100% response rate (260 responses), the follow-up survey only accounted for 95.4% (248 responses). Therefore, we only used the results from the 248 responses (129 from the intervention group and 119 from the comparison group) in our analysis. Children and parents in the intervention group took part in the eye health intervention education curriculum for four weeks, while the comparison group did not. This study was approved by the institutional review board of Research Ethics Committee (REC) of the National Taiwan Normal University (REC No. 201802HS004).

### 2.4. Measurements

Questionnaires were used to collect children’s and parents’ eye health knowledge, beliefs, cues to action, self-efficacy, and behaviors (please see Supplementary Materials, Survey Questionnaire). The questionnaires were developed using the key concepts of the HBM [32], parental involvement [36], and the eye health intervention curriculum developed for this study. The children’s questionnaires used words and pictures to aid children’s understanding of the questions. Six expert reviewers were invited to assess the content validity of the eye health intervention education curriculum and evaluation questionnaires. Content validity indices of the children’s and parent’s questionnaires were 0.89 and 0.92, which indicates a slight difference between the children’s and their parent’s responses. The curriculum and questionnaires were revised according to the reviewer’s comments following the content validity assessment. Subsequently, pre-test surveys among 40 parent–child groups were conducted to examine their responses and to evaluate the reliability of the data. The pre-test survey found that the internal consistency of the questionnaire items was within the acceptable limit (Cronbach’s α ≥ 0.6).

#### 2.4.1. Eye Health Knowledge

Based on the HBM , children’s eye health knowledge was comprised of four items: (1) screen use time every day, (2) screen use time every time, (3) screen use distance, and (4) outdoor activities. A sample question was, “How long do you think your screen time should be every day?” There were three response options for each item: (a) which is the most correct answer; (b) an incorrect answer; and (c) “unknown”. Each correct response scored 1 point, while each incorrect or “unknown” response scored 0.

There were 10 question items related to eye health knowledge that required the parent’s response. A sample question is as follows: “Which of the following is the most important behavioral factor leading to myopia” Each correct response scored 1 point, while each incorrect or “unknown” response scored 0.

#### 2.4.2. Eye Health Beliefs

Children were asked eight questions about eye health beliefs, including perceived susceptibility (one item), perceived severity (one item), perceived benefits (three items), and perceived barriers (three items). An example is as follows: “Do you think it is serious that screen overuse makes your eyes uncomfortable?” The response included two options for perceived severity from cartoon images: “Not serious” (1) and “Serious” (2). A higher score indicated better awareness of eye health practices. Cronbach’s α for the children’s eye health beliefs scale was 0.72.

Parents were asked to respond to 16 questions related to eye health beliefs, including perceived susceptibility (three items), perceived severity (four items), perceived benefits (three items), and perceived barriers (six items). A sample question is as follows: “I think that children who often watch electronic products can easily lead to premature myopia (eye strain).” The response options for each item were evaluated using a five-point Likert scale that ranged from “Strongly disagree” (1) to “Strongly agree” (5). After reversing the perceived barriers’ scores, the sum of all domains was the total score of parents’ eye health beliefs. A higher score indicated a higher level of parental eye health beliefs. Cronbach’s α for the parent’s eye health beliefs scale was 0.86.

#### 2.4.3. Eye Health Cues to Action

There were three items associated with cues to action in which the children were asked to respond. A sample question was, “Has your teacher ever taught you how to protect your eyes when using a screen?” This was a yes or no response and the scoring was a 1 (yes) or 0 (no). A higher score indicated a higher level of awareness of cues for maintaining eye health. The Cronbach’s α of the children’s eye health cues to action scale was 0.60.

Parents were asked to respond to four cues to action questions. A sample question was, “The preschool teacher has told me how to protect my child’s eye when using the screen.” There were two available options (answers), “Yes” and “No”, which were coded as “1” and “0”, respectively. A higher score indicated a higher level of parental eye health cues to action. The Cronbach’s α of the parents’ eye health cues to action scale was 0.86.

#### 2.4.4. Eye Health Self-Efficacy

The children’s eye health self-efficacy contained four questions to assess their confidence level. A sample question was, “Do you feel confident that you use screen no more than one hour a day?” Each of these items had two available options (answers), “Confident” (coded as “1”), and “Not confident” (coded as “0”) from cartoon images. A higher score indicated a higher level of awareness of eye health self-efficacy. Cronbach’s α for the children’s eye health self-efficacy scale was 0.79.

There were 11 questions related to parent’s eye health parenting efficacy. A sample question was, “I can remind my child to keep a proper distance when they use the screen.” A five-pint Likert scale measurement (i.e., Not very confident = 1; Very confident = 5) was used for each of these items according to a rating of their confidence ratio from 0 to 100%. A higher score indicated a higher level of eye health parenting efficacy. Cronbach’s α for the parent’s eye health parenting efficacy scale was 0.83.

#### 2.4.5. Eye Health Behaviors

There were four questions about eye health behaviors that the children were asked for a response. An example is as follows: “Do you feel confident that your screen use is no more than one hour a day?” A three-point Likert scale (“Never” = 1; “Sometimes” = 2; “Always” = 3) was used to evaluate their responses by cartoon images. A higher score indicated a higher frequency of children’s eye health behaviors. The Cronbach’s α of the children’s eye health behavior scale was 0.70.

Parent’s eye health parenting behaviors consisted of 11 questions. A sample is as follows: “I can remind my child to keep a proper distance when he/she uses the screen.” The response for each item was measured through a five-point Likert scale (“Never” = 1; “Always” = 5). A higher score indicated a higher frequency of parents’ eye health parenting behaviors. The Cronbach’s α of the parents’ eye health parenting behavior scale was 0.88.

### 2.5. Socio-Demographic Characteristics

Children’s socio-demographic characteristics in the present study included gender, time spent using screens every day (i.e., less than one hour, one hour or more), and children’s vision (i.e., myopic, not myopic, or do not know), as reported by their parents. The parent’s socio-demographic characteristics included parental role (father or mother), age (under 37 or over 37), education level (high school or below, college or university, or postgraduate), and vision (myopic, not myopic or unknown).

### 2.6. Data Analysis

Statistical analyses were performed using SPSS version 23.0 (IBM, Armonk, NY, USA). Descriptive statistics were calculated for all variables, whereas chi-square tests were conducted to examine background variables between the intervention and comparison groups at baseline. In addition, the generalized estimating equation (GEE) approach was used to examine the effects of the intervention on children’s and parental eye health knowledge, health beliefs, cues to action, self-efficacy, and behaviors.

## 3. Results

### 3.1. Socio-Demographic Characteristics

Our analysis was based on the responses from 248 parent–child pairs, comprised of 129 (52%) parent–child pairs from the intervention group and the remaining pairs (*n* = 119, 48%) from the comparison group. Children’s gender was equally represented with males and females of 127 (51.2%) and 121 (48.8%), respectively. The majority (*n* = 194, 78.2%) of the children indicated that they had myopia, with 39 (15.7%) responding as unknown (*n* = 39, 15.7%) and 15 (6.1%) responding as being nonmyopic. Although children’s screen use time was very similar between less than one hour (*n* = 123, 49.6%) and one or more (*n* = 125, 50.4%) hours during the weekdays, but during the weekends 66.1% (*n* = 164) of the children would spend one hour or more on screen use time as compared to 33.9% (*n* = 84) who spent less than one hour.

A vast majority considered their parental role as “Mother” (*n* = 189, 76.2%); the parental role of a “Father” accounted for 23.8% (*n* = 59). In terms of age group, parents were equally represented in the categories: younger than 37 years old (*n* = 121, 48.8%) and 37 years and older (*n* = 127, 51.2%) because their average age was 37 years old. More than half of the parents had attained a college or university qualification (*n* = 138, 55.7%), and this was followed by postgraduate (*n* = 75, 30.2%), and high school or below (*n* = 35, 14.1%) qualifications. Parents who had myopia accounted for 64.5% (*n* = 160) with the remaining 35.5% (*n* = 88) being nonmyopic.

The chi-square test results revealed no significant differences between the intervention and comparison groups on the children’s and parent’s socio-demographic characteristics. Table 1 below provides a summary of the socio-demographic characteristics of the intervention and comparison groups for children and their parents.

### 3.2. Changes in Children’s Outcome Variables

The mean score for the children’s eye health knowledge in the intervention group (0.63) was lower than the comparison group (0.66) at the baseline survey (Table 2). However, the mean score in the intervention group improved to 0.97 at the follow-up survey and was higher than the comparison group which remained about the same at 0.68. In terms of the children’s eye health benefits, the mean scores for the intervention and comparison groups were similar at 1.64 and 1.67, respectively, at the baseline survey. At the follow-up survey, the mean score for the intervention group had increased to 1.98, which was higher than the comparison group’s score of 1.69.

For the children’s eye health cues to action, the intervention group’s mean score was 0.39 as compared to the comparison group of 0.57 at the baseline survey. However, at the follow-up survey, the mean score for the intervention group increased to 0.78, which was higher than the comparison group score of 0.51. With regards to the children’s eye health self-efficacy, both the intervention and comparison groups had a mean score of 1.69 at the baseline survey. However, the mean score for the intervention group increased to 1.98 at the follow-up survey, which was higher than the comparison group’s score of 1.68. The mean scores for the children’s eye health behaviors were 1.52 and 1.49, respectively, for the intervention and comparison groups at the baseline survey. The intervention group’s mean score increased to 1.91 at the follow-up survey, which was higher than the comparison group’s score of 1.35.

Our findings also indicated that there was a consistent improvement in the intervention group’s mean score for all the items in the children’s eye health knowledge, beliefs, cues to action, self-efficacy, and behaviors when comparing the baseline and follow-up survey scores. Table 2 presents the summary findings of the baseline and follow-up surveys for the children’s eye health knowledge, beliefs, cues to action, self-efficacy, and behaviors between the intervention and comparison groups.

### 3.3. Changes in Parents’ Outcome Variables

Our findings showed that the mean scores in the baseline survey for parent’s eye health knowledge in both the intervention and comparison groups were comparable at 0.69 and 0.70, respectively. For the follow-up survey, the mean score for the intervention group increased to 0.76, and the comparison group’s score also increased to 0.72. For the parent’s eye health beliefs, the mean score for the intervention group was 4.10 at the baseline survey, which was higher than the comparison group’s score of 4.07. At the follow-up survey, the mean scores for both groups increased to 4.23 and 4.11, respectively.

For the parent’s eye health cues to action, the intervention group’s score was 0.41, which was lower than the comparison group score of 0.46 for the baseline survey. However, the intervention group’s mean score improved to 0.65 in the follow-up survey and was higher than the comparison group’s score of 0.51. In terms of the parents’ eye health parenting efficacy, the mean scores for the intervention and comparison groups were 3.95 and 4.04, respectively, for the baseline survey. The intervention group’s mean score increased to 4.19 while the comparison group’s score was 4.11 at the follow-up survey. At the baseline survey, the mean score for the parents’ eye health parenting behaviors in the intervention group’s score was lower than that of the comparison group; 4.09 versus 4.19. The intervention group’s mean score increased to 4.18 at the follow-up survey, which was similar to the comparison group’s score of 4.17.

The results also revealed that the mean scores for the parents’ eye health knowledge, beliefs, cues to action, parenting efficacy, and parenting behaviors improved from the baseline to the follow-up survey (Table 3).

### 3.4. Effects of the Intervention on Outcome Indicators

As shown in Table 4, the findings from the GEE analysis revealed that the implementation of a preschool-based eye health intervention program with parental involvement had positive effects on the increasing level of eye health knowledge (β = 0.32, *p* < 0.001), beliefs (β = 0.29, *p* < 0.001), cues to action (β = 0.44, *p* < 0.001), self-efficacy (β = 0.30, *p* < 0.001), and behaviors (β = 0.53, *p* < 0.001) in children. On the other hand, the intervention program had positive effects on improving eye health knowledge (β = 0.04, *p* < 0.03), cues to action (β = 0.18, *p* < 0.001), and parenting efficacy (β = 0.16, *p* < 0.02) for the parent group (please refer to Table 5).

## 4. Discussion

The results of this study from a Chinese preschool-based eye health intervention program with parental involvement showed effective enhancement of children’s eye health knowledge, beliefs, cues to action, and behaviors. These findings were consistent with a study conducted in Vietnam [24] about the effects of school eye health promotion on children’s eye health literacy. Parental involvement has been widely recognized as a vital strategy for improving children’s physical health, behavior, and mental development in general [30,37,38,39,40], and parental involvement intervention programs have been known to be effective in reducing children’s screen use time [41]. This was further supported by a study [26] revealing that parents who had higher levels of risk perception and parental efficacy were more likely to positively reduce children’s screen time and improve their eye health behaviors. Previous preschool-based intervention studies had also indicated that educating parents could potentially increase parents’ eye health behaviors and rates of eye examinations among preschool children [42]. Therefore, strengthening the capacities and capabilities of preschool teachers and implementing eye health education programs or courses that involved parents should be considered in order to improve children’s eye health.

This study found that children rarely understood the benefits of outdoor activities for the prevention of myopia, while previous studies [43,44,45] suggested that increasing the duration of outdoor activities could possibly reduce the incidence and development of myopia. Other eye health prevention studies [46,47,48] had also indicated that two or more hours a day spent on outdoor activities could have a protective effect on children’s vision. The Ministry of Education (People’s Republic of China) had actively promulgated the importance of this through the Guide to Learning and Development for Children Aged 3–6 Years, which stipulated that children should spend at least two hours a day on outdoor activities [49]. However, the results of the present study showed that participating parents had lower scores in terms of their eye health parenting knowledge, beliefs, efficacy, and parenting behaviors about outdoor activities, which could be a barrier to encouraging children to spend more time on outdoor activities. Furthermore, the 2018 World Health Organization Meeting on Myopia Control recommended that outdoor time of 2–3 h per day as a practical public health intervention for school children [50]. This evidence indicated the need to increase public awareness (e.g., through eye health promotion campaigns by the government) toward the benefits of two or more hours of outdoor activities per day on eye health, which could potentially enhance children’s and parents’ beliefs about outdoor activity efficacy.

Findings from this study also revealed that children had attained lower scores on their beliefs and self-efficacy in the control of screen time. In terms of eye health self-efficacy, more than 40% of the children indicated that they had no confidence in themselves to control their screen time. This was also consistent with the low level of beliefs in their ability to control screen time, whereby more than half of the children spent one or more hours on screen. However, the recommendation by the American Academy of Pediatrics suggested that children should not spend more than one hour per day on screen [12] because this could result in eyestrain symptoms [19]. In addition, an experimental study in China showed that prolong screen use time could significantly affect school children’s vision [51]. On the other hand, parents in this study reported increasing difficulty to control their children’s screen use and engaged in outdoor activities. This could be explained by the fact that most dual-employed parent households were busy with work [52] and spent little time with their children on outdoor activities. Thus, educational workshops about intervention techniques and strategies could be conducted to help children and parents to better manage the screen time and usage frequencies. Work–life–health balance should also be encouraged by government agencies and nongovernmental organizations to enable working parents to spend more time with their children in order to minimize screen time and participate in more outdoor activities.

The present study revealed that while a preschool-based eye health intervention program combined with parental involvement had significantly improved parents’ knowledge, cues to action, and parental efficacy, their health beliefs and parenting behaviors had not shown much of a similar result. One possible reason could be the high level of eye health beliefs for both the intervention and comparison parent groups in the baseline and follow-up surveys, whereby significant improvements were unnoticeable. Although some studies indicated that barriers and misconceptions about eye health among parents were key issues that need to be addressed [53,54], others found that participants’ health beliefs [35] or behaviors [55] did not increase significantly after the educational intervention. Given that the intervention period of this study was approximately one month, only short-term effects were evaluated. An extended evaluation period could provide greater insights to the extent of the effects the educational intervention program have on improving parents’ eye health knowledge, cues to action, and efficacy improves parenting behaviors.

## 5. Limitations and Future Research

There are a few limitations in this study. Firstly, due to the cognitive ability of young children, they had to be assessed through group interviews by the researchers. Thus, the reliability of the results of the children’s questionnaires may have been affected. However, a pilot test was conducted, and revisions were made to the questionnaire based on the children’s responses and comprehension abilities. In addition, during the interview process, researchers had taken the time to explain (combined with pictures) the questions to the children, repeated and elaborated them (if necessary), to ensure their understanding.

Secondly, this study only involved educational intervention in a large public preschool consisting of five branches in Beijing; therefore, the external validity is limited. Future research can be extended to other preschools in different regions, and the results can only be compared and generalized to a wide range of preschool groups in China. However, this study can also be replicated in other countries to determine similarities and differences. Finally, this study implemented a four-week eye health intervention program that focused on the short-term evaluation of the effects on parents’ and children’s eye health knowledge, beliefs, cues to action, self-efficacy, and behaviors. Future research can consider assessing the impact of these factors on a longer-term basis and examine how they could potentially delay and control the development of myopia in children.

## 6. Conclusions

In conclusion, this study has used the Health Belief Model to investigate the children’s and their parents’ eye health knowledge, beliefs, cues to action, self-efficacy, and behaviors with an eye health intervention education program. Results showed that eye health intervention on screen use with parental involvement had a positive effect through an increase in eye health knowledge, beliefs, cues to action, self-efficacy, and behaviors among children. The eye health intervention also had positive effects on the parents with results showing improved eye health knowledge, cues to action, and parenting efficacy. Findings from this study indicated the need for government and nongovernmental organizations to collaborate toward promoting a more comprehensive school-based eye health intervention program, strengthening the capacities and capabilities of preschool teachers to support the implementation of these programs, and outdoor activity policies [56,57] in order to improve children’s eye health.

## Figures and Tables

**Table 1:** Socio-demographic characteristics of intervention and comparison groups for children and parents.

	Intervention Group		Comparison Group		Chi-Square	*p* Value
	*n*	%	*n*	%		
**Children**						
Gender					1.35	0.246
Male	61	47.3	66	55.5		
Female	68	52.7	53	44.5		
Myopia					3.53	0.171
Yes	11	8.5	4	3.4		
No	96	74.4	98	82.4		
Unknown	22	17.1	17	14.3		
Screen use time every weekday					0.78	0.376
<1 h	60	46.5	63	52.9		
≥1 h	69	53.5	56	47.1		
Screen use time every weekend					0.05	0.829
<1 h	45	34.9	39	32.8		
≥1 h	84	65.1	80	67.2		
**Parents**						
Parental role					0.70	0.402
Father	34	26.4	25	21.0		
Mother	95	73.6	94	79.0		
Age (average age = 37 years old)					0.00	1
<37 years old	63	48.8	58	48.7		
≥37 years old	66	51.2	61	51.3		
Education					1.29	0.525
High school or below	16	12.4	19	16.0		
College or university	76	58.9	62	52.1		
Postgraduate	37	28.7	38	31.9		
Myopia					1.58	0.209
Yes	78	60.5	82	68.9		
No	51	39.5	37	31.1		

**Table 2:** Children’s eye health knowledge, beliefs, cues to action, self-efficacy, and behaviors for the intervention and comparison groups based on the baseline and follow-up surveys.

	Intervention Group				Comparison Group			
	Baseline		Follow-Up		Baseline		Follow-Up	
	Mean	SD	Mean	SD	Mean	SD	Mean	SD
Eye health knowledge	0.63	0.23	0.97	0.10	0.66	0.19	0.68	0.17
Screen time everyday	0.77	0.42	0.96	0.19	0.79	0.41	0.84	0.37
Screen time every time	0.74	0.44	0.97	0.17	0.76	0.43	0.80	0.40
Screen use distance	0.78	0.42	1.00	0.00	0.84	0.37	0.86	0.35
Outdoor activities	0.24	0.43	0.95	0.23	0.25	0.44	0.22	0.41
Eye health beliefs	1.64	0.23	1.96	0.10	1.67	0.21	1.69	0.23
Perceived susceptibility	1.78	0.42	1.91	0.28	1.77	0.42	1.76	0.43
Perceived severity	1.64	0.48	1.95	0.21	1.82	0.38	1.65	0.48
Perceived benefits	1.58	0.33	1.98	0.09	1.56	0.34	1.65	0.34
Perceived barriers	1.66	0.34	1.95	0.14	1.69	0.32	1.71	0.32
Eye health cues to action	0.39	0.29	0.78	0.22	0.57	0.28	0.51	0.31
Get messages from teachers	0.24	0.43	0.98	0.15	0.57	0.50	0.46	0.50
Get messages from parents	0.66	0.48	0.84	0.36	0.74	0.44	0.67	0.47
Get messages from ophthalmologists	0.28	0.45	0.51	0.50	0.39	0.49	0.40	0.49
Eye health self-efficacy	1.69	0.32	1.98	0.10	1.69	0.32	1.68	0.33
Screen time everyday	1.72	0.45	1.98	0.15	1.65	0.48	1.69	0.46
Screen time every time	1.64	0.48	1.98	0.15	1.67	0.47	1.69	0.46
Screen use distance	1.73	0.45	1.98	0.12	1.77	0.42	1.74	0.44
Outdoor activities	1.66	0.48	1.97	0.17	1.67	0.47	1.61	0.49
Eye health behaviors	1.52	0.52	1.91	0.26	1.49	0.48	1.35	0.55
Screen time everyday	1.55	0.71	1.94	0.30	1.50	0.75	1.38	0.84
Screen time every time	1.49	0.74	1.88	0.43	1.58	0.69	1.39	0.85
Screen use distance	1.63	0.66	1.94	0.32	1.53	0.72	1.50	0.80
Outdoor activities	1.40	0.78	1.87	0.44	1.37	0.82	1.13	0.91

**Table 3:** Parents’ eye health knowledge, beliefs, cues to action, parenting efficacy, and parenting behaviors for the intervention and comparison groups based on the baseline and follow-up surveys.

	Intervention Group				Comparison Group			
	Baseline		Follow-Up		Baseline		Follow-Up	
	Mean	SD	Mean	SD	Mean	SD	Mean	SD
Eye health knowledge	0.69	0.16	0.76	0.17	0.70	0.16	0.72	0.15
Cause of myopia	0.76	0.27	0.83	0.25	0.73	0.29	0.80	0.25
Risk factor of myopia	0.45	0.25	0.59	0.32	0.50	0.24	0.48	0.26
Eye health behaviors	0.75	0.20	0.79	0.18	0.76	0.17	0.78	0.17
Eye health beliefs	4.10	0.49	4.23	0.54	4.07	0.52	4.11	0.52
Perceived susceptibility	4.57	0.54	4.62	0.65	4.54	0.78	4.63	0.68
Perceived severity	4.61	0.56	4.64	0.63	4.57	0.70	4.62	0.70
Perceived benefits	4.57	0.57	4.65	0.63	4.61	0.64	4.59	0.71
Perceived barriers	3.31	0.92	3.54	0.88	3.22	0.99	3.28	0.86
Eye health cues to action	0.41	0.28	0.65	0.31	0.46	0.28	0.51	0.28
Participation in educational courses	0.08	0.27	0.36	0.48	0.12	0.32	0.14	0.35
Get messages from the internet	0.26	0.44	0.60	0.49	0.30	0.46	0.45	0.50
Get messages from the teacher	0.76	0.43	0.91	0.28	0.78	0.41	0.81	0.40
Get messages from the ophthalmologist	0.57	0.50	0.71	0.46	0.65	0.48	0.66	0.48
Eye health parenting efficacy	3.95	0.74	4.19	0.68	4.04	0.58	4.11	0.61
Distance and poster	4.25	0.86	4.36	0.75	4.36	0.63	4.37	0.70
Time management	3.95	1.06	4.28	0.82	4.03	0.92	4.16	0.77
Environment	4.11	0.81	4.31	0.74	4.25	0.65	4.24	0.68
Outdoor activities	3.53	1.15	3.87	0.98	3.59	1.03	3.77	1.00
Vision examination	3.25	1.09	3.57	1.08	3.24	1.16	3.44	1.11
Family rule	3.87	1.09	4.10	0.95	3.83	1.10	3.97	1.00
Eye health parenting behaviors	4.09	0.69	4.18	0.74	4.19	0.56	4.17	0.57
Distance and posture	4.28	0.82	4.29	0.90	4.40	0.66	4.33	0.71
Time management	4.17	0.95	4.20	0.98	4.24	0.81	4.23	0.80
Environment	4.20	0.73	4.27	0.79	4.31	0.66	4.22	0.67
Outdoor activities	3.91	0.95	4.06	0.88	3.94	0.81	3.95	0.80
Vision examination	3.40	1.03	3.70	0.97	3.62	0.98	3.76	0.92
Family rule	4.02	0.98	4.14	0.97	3.99	0.90	4.10	0.84

**Table 4:** Multivariate analysis of children’ eye health knowledge, beliefs, cues to action, self-efficacy, and behaviors.

	β	SE	*p* Value
Eye health knowledge			
Intercept	0.66	0.02	<0.001
Time	0.02	0.02	0.350
Group	−0.03	0.03	0.260
Time × Group	0.32	0.03	<0.001
Eye health beliefs			
Intercept	1.67	0.02	<0.001
Time	0.02	0.02	0.330
Group	−0.02	0.03	0.380
Time × Group	0.29	0.03	<0.001
Eye health cues to action			
Intercept	0.57	0.03	<0.001
Time	−0.06	0.02	0.020
Group	−0.18	0.04	<0.001
Time × Group	0.44	0.03	<0.001
Eye health self-efficacy			
Intercept	1.69	0.03	<0.001
Time	−0.01	0.02	0.720
Group	0.00	0.04	0.940
Time × Group	0.30	0.04	<0.001
Eye health behaviors			
Intercept	1.49	0.04	<0.001
Time	−0.14	0.04	0.060
Group	0.02	0.06	0.890
Time × Group	0.53	0.06	<0.001

Notes: *n* = 248, intervention group: *n* = 129, comparison group: *n* = 119. The generalized estimating equation analysis was used. Y = β0 + β1 (time) + β2 (group) + β3 (time × group); Y = children’ eye health knowledge/beliefs/cues to action/self-efficacy/behaviors regarding screen use. Time: baseline = 0, follow-up = 1; Group: comparison group = 0, intervention group = 1.

**Table 5:** Multivariate analysis of parents’ eye health parenting knowledge, beliefs, cues to action, parenting efficacy, and parenting behaviors.

	β	SE	*p* Value
Eye health knowledge			
Intercept	0.70	0.01	<0.001
Time	0.02	0.01	0.120
Group	−0.01	0.02	0.560
Time × Group	0.04	0.02	0.030
Eye health beliefs			
Intercept	4.07	0.05	<0.001
Time	0.05	0.04	0.230
Group	0.04	0.06	0.560
Time × Group	0.07	0.05	0.160
Eye health cues to action			
Intercept	0.46	0.03	<0.001
Time	0.05	0.03	0.080
Group	−0.05	0.04	0.180
Time × Group	0.18	0.04	<0.001
Eye health parenting efficacy			
Intercept	4.04	0.05	<0.001
Time	0.07	0.04	0.090
Group	−0.09	0.08	0.290
Time × Group	0.16	0.07	0.020
Eye health parenting behaviors			
Intercept	4.19	0.05	<0.001
Time	−0.02	0.04	0.640
Group	−0.09	0.08	0.240
Time × Group	0.10	0.07	0.150

Notes: *n* = 248, intervention group: *n* = 129, comparison group: *n* = 119. The generalized estimating equation analysis was used. Y = β0 + β1 (time) + β2 (group) + β3 (time × group); Y = Parents’ eye health parenting knowledge/beliefs/cues to action/parenting efficacy/behaviors regarding children’s screen use. Time: baseline = 0, follow-up = 1; Group: comparison group = 0, intervention group = 1.

## Data Availability

The study did not report any data.

## Supplementary Materials

The following are available online at https://www.mdpi.com/article/10.3390/ijerph182111330/s1, a Survey Questionnaire.
