# Supplementary material for: Effects of Parental Involvement in a Preschool-Based Eye Health Intervention Regarding Children’s Screen Use in China
Source: Int J Environ Res Public Health. 2021 Oct 28;18(21):11330. doi: 10.3390/ijerph182111330 (PMC8582736; doi:10.3390/ijerph182111330)
Supplement: Supplementary file 1 [file ijerph-18-11330-s001.zip › ijerph-1397767-supplementary.pdf]

International Journal of
Environmental Research and Public Health

MDPI

Survey Questionnaire

A. Questionnaire introduction and informed consent for study participants

Dear Parents,

This is a survey is supported by the Cultivation Project of Zhejiang Provincial Advantageous and Characteristic Disciplines in the Jing Hengyi College of Education of Hangzhou Normal University (No.19JYXK005), the Zhejiang Provincial Educational Science Planning Research Subject (No. 2020SCG012), the research funds of Hangzhou Normal University (No. RWSK20200406), and the Taiwan Ministry of Science and Technology (MOST 104-2511-S-003-009-MY2). The main objective of this questionnaire is to gain a better understanding on the effects of parental involvement in a preschool-based eye health intervention regarding your children's screen use looking at the mobile phone or phablet. Participants are deemed to have agreed and provided consent to participate in the survey when they successfully completed and submitted to us. Please note that each questionnaire can take approximately 15 min for you and for your kids studying in kindergarten to complete. Thank you for taking the time to complete it.

This questionnaire has been designed to be completed and will identify individual participants because we will test twice. The research team will take all reasonable steps to maintain your privacy and the confidentiality of the data collected. This research results will be published in academic journals with findings reported as a summary where no individuals will be identified. There are no commercial benefits derived from this study.

Please do not feel obliged to participate in this study; however, once you have started and submitted your and your kid's answers, you are deemed to agree to participate. Because this questionnaire will be pre-coded for your identification purpose to retest from a test–retest method, the research team will not be able to delete your questionnaires and their contents once they have been filled out and submitted to us as well as a follow-up survey.

If you wish to inquire about the content of this questionnaire, or are interested in knowing the results of the research, you are welcome to request a summary of the research results by contacting (Project advisor: Fong-Ching Chang, Tel: +886-2-77491711, email: fongchingchang@ntnu.edu.tw) at the Department of Health Promotion and Health Education, National Taiwan Normal University (NTNU). This study was approved (REC No. 201802HS004) by the institutional review board of Research Ethics Committee (REC) of the NTNU.

Project PI: Shu-Mei Liu
Jing Hengyi College of Education, Hangzhou Normal University
Project advisor: Fong-Ching Chang
Department of Health Promotion and Health Education, NTNU

B. Basic information

1. Your child's gender:
□male □female
2. Does your child have myopia (nearsightedness) by ophthalmologists (eye doctors)?
□yes □no □unknown
3. How many days did your child watch electronic products while eating during last week?
□0 days □1 day □2 days □3 days □4 days □5 days □6 days □7 days
4. How many days did your child spend watching electronic products at night before going to bed during last week?
□0 days □1 day □2 days □3 days □4 days □5 days □6 days □7 days
5. During last week, the average time your child spent watching electronic products on weekdays (Monday to Friday):
____h _____ minute/day
6. During last week, the average time your child spent watching electronic products on holidays (Saturday and Sunday):
____ h_____ minute/day

7. Your parental role:
□father □mother
8. Your age _______________
9. Your education qualification:
□ high school or below □ college or university (undergraduate) □ postgraduate
10. Does you have myopia (nearsightedness)?
□yes □no

C. Questions for your child looking at the mobile phone or phablet (for children role only) (No. __) (for authorized attendees only)

1. How long do you think your screen time should be every day?

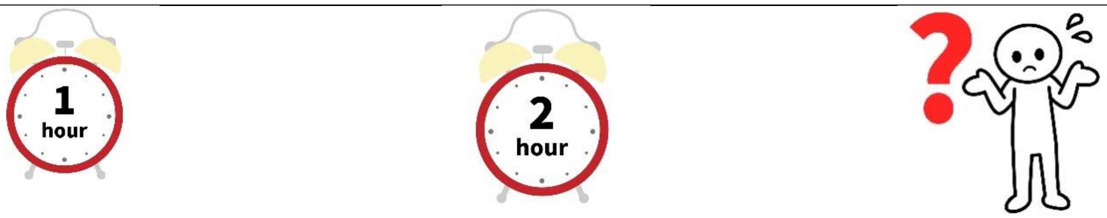

2. How long do you think your screen time should be every time?

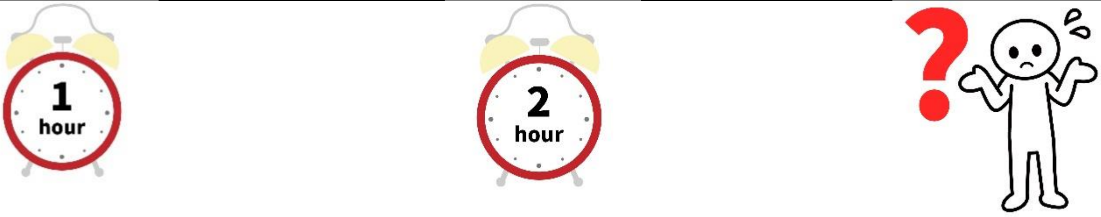

3. When looking at screens, how far away is appropriate?

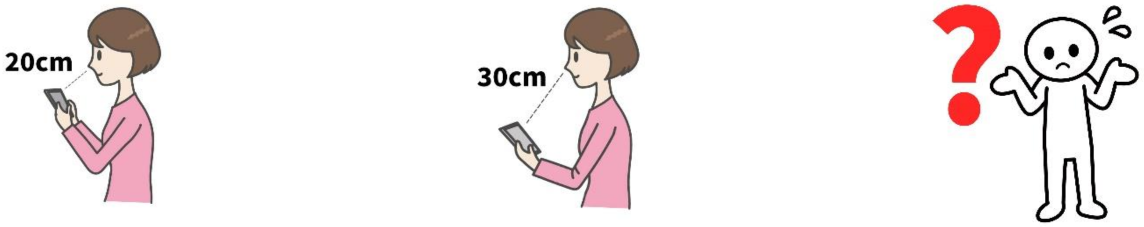

4. At least, how long is it, to stay out of myopia every day to play outdoor activities?

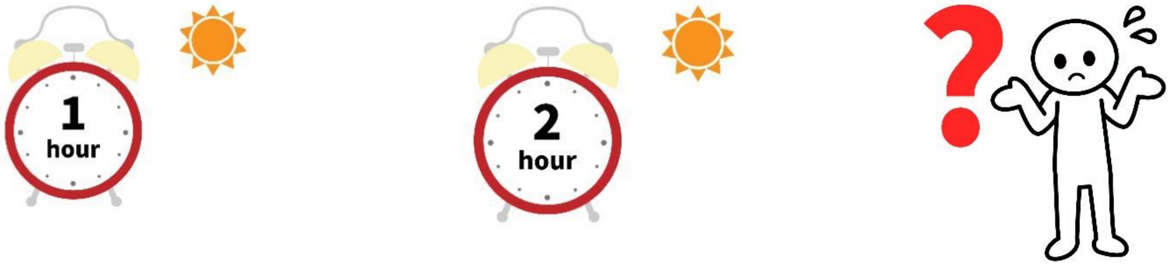

5. Do you think that using screens often makes your eyes uncomfortable?

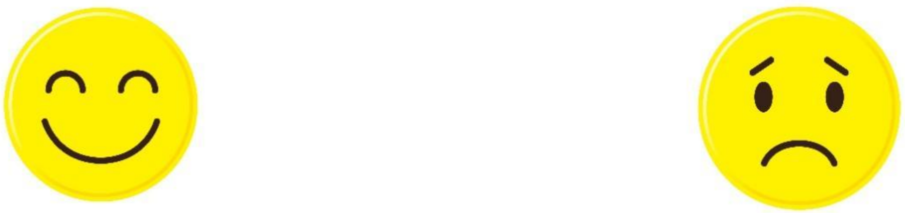

6. Do you think it is serious that screen overuse makes your eyes uncomfortable?

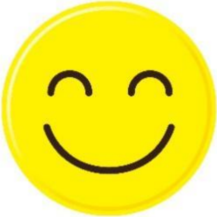
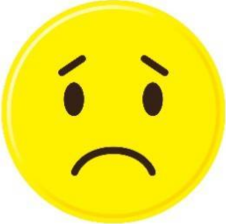

7. Do you think that restricting screen uses no more than one hour a day is good for your eyes?

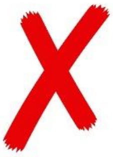
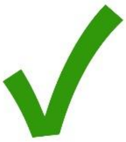

8. Do you think that restricting screen uses no more than half an hour a day is good for your eyes?

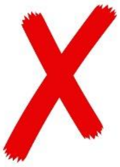
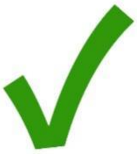

9. Do you think that playing outdoors for more than two hours a day is good for eyes?

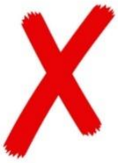
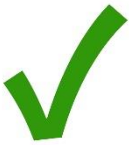

10. Do you think it is hard for you to restrict screen uses no more than one hour per day?

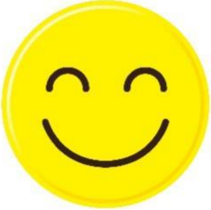
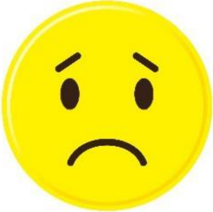

11. Do you think it is hard for you to restrict screen uses no more than half an hour per day?

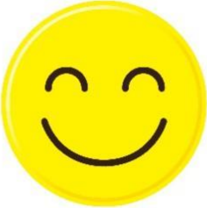 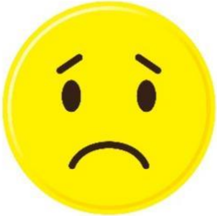

12. Do you think it is hard for you to spend more than two hours outside every day?

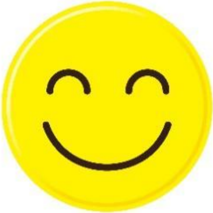 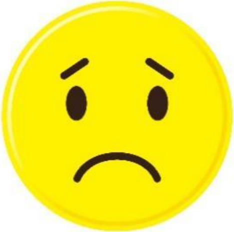

13. Has your teacher ever taught you how to protect your eyes when using a screen?

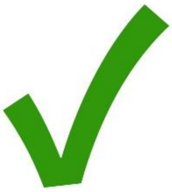 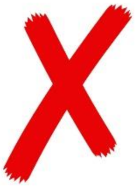

14. Has your family taught you how to protect your eyes when using a screen?

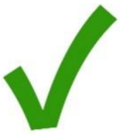 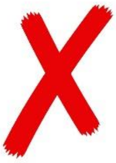

15. In the last month, did your family take you to the eye doctors to have your eyes checked?

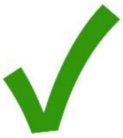 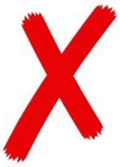

16. Do you feel confident that your screen use is no more than one hour a day?

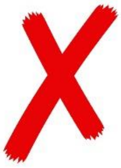 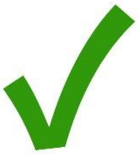

17. Do you feel confident that your screen use is no more than half an hour a day?

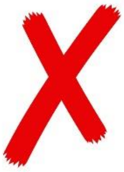 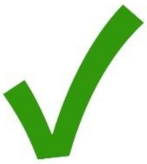

18. Do you feel confident that you use screen to keep a proper distance?

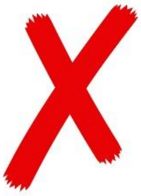 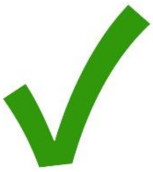

19. At the weekends or during the holidays, do you feel confident that you spend more than two hours playing outdoor activities every day?

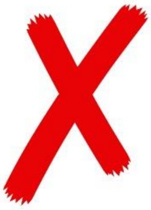 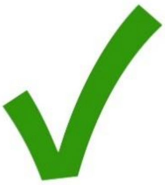

20. In the last week, have you use any screens within an hour per day?

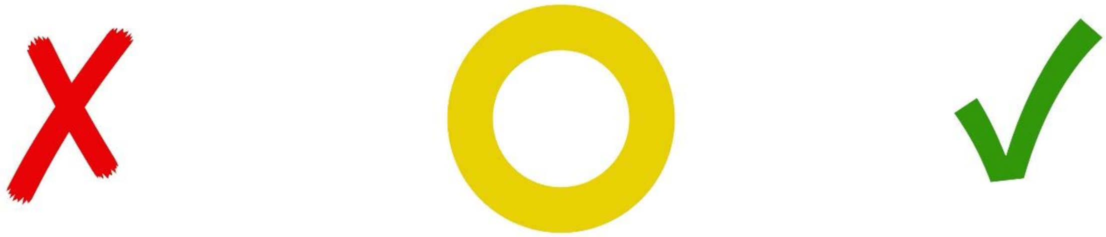

21. In the last week, have you use any screens within half an hour per day?

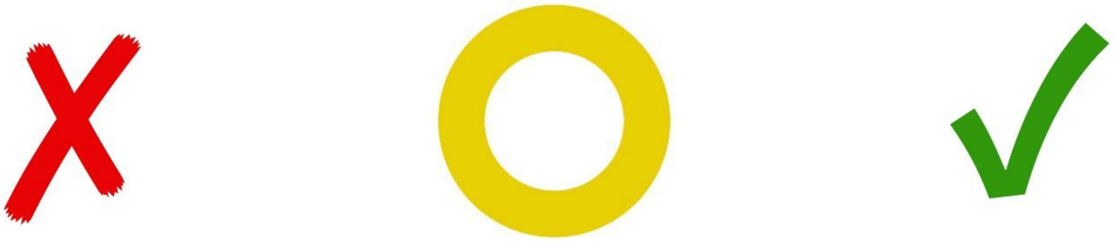

22. In the last week, have you use any screens to keep a proper distance?

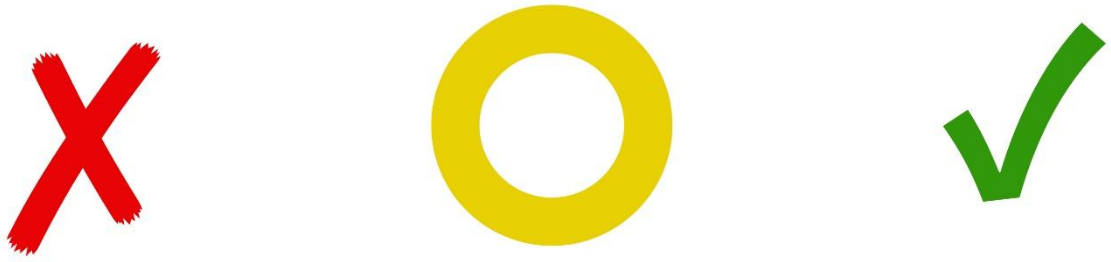

23. In the last week, have you spent more than two hours playing outdoor activities every day?

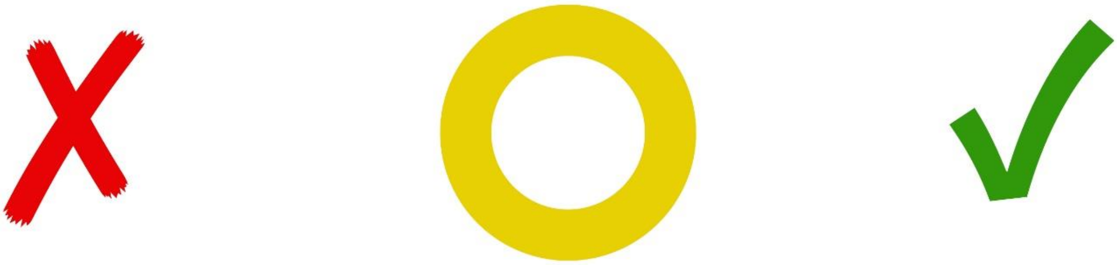

D. Questions for you (for parental role only) (No. __) (for authorized attendees only). Please read all of the instructions. If you have any trouble with your test or understanding test questions, contact your instructor.

Question sets D1 (10 items):<br>Answer instructions: We wish to understand your perception of your child's vision care in using electronic products. Please read the questions carefully and select the most suitable answer for each question.

1. Which of the following is the most important behavioral factor leading to myopia?
□ (1) Excessive use of eyes at close range for a long time
□ (2) Frequently rub your eyes with your hands
□ (3) Not taking in balanced nutrition
□ (4) Irregular sleep
□ (5) Don't know

2. Regarding myopia, which of the following statements is wrong?
□ (1) Myopia is caused by the elongated eye axis; when looking at distant objects, it cannot be imaged on the retina
□ (2) Myopia is a disease
□ (3) After the eye axis is elongated, it can be restored
□ (4) When children have blurred vision, it may be pseudo-myopia
□ (5) Don't know

3. Which of the following statement is not included in the complications that are prone to high myopia?
□ (1) Glaucoma
□ (2) Cataract
□ (3) Leukoplakia
□ (4) Retina degeneration or detachment
□ (5) Don't know

4. Which of the following statement is correct about the blue light of electronic products?
□ (1) Adult eyeballs have matured, and blue light from electronic products will not harm adult eyes
□ (2) Exposure to blue light for a long time may cause damage to the retina of the eye
□ (3) The blue light emitted by mobile phones and tablets is very weak, so you don't have to worry about hurting your eyes
□ (4) Put on an anti-blue film or wear anti-blue glasses, so you don't have to worry about the impact of using electronic products on your eyesight
□ (5) Don't know

5. How often does a child's vision health check take?
□ (1) If the vision is normal, there is no need for regular vision checks
□ (2) Check once every six months, there should be twice a year
□ (3) Inspection once a year
□ (4) Inspection once every two years
□ (5) Don't know

6. Which of the following behaviors when viewing electronic products is beneficial to vision care?
□ (1) Can be seen on a car with enough light
□ (2) Can lie down and watch in a comfortable position on the bed
□ (3) Looking under the heavy sun
□ (4) When the room is dim, turn on the headlights and desk lamps at the same time

□ (5) Don't know

7. After reading for a long time, which of the following methods can relieve fatigue and get the best rest?
□ (1) You can relax your eyes by watching TV
□ (2) You can play outdoors for a while
□ (3) You can rub your eyes with both hands
□ (4) You can play a game on the phone for a while
□ (5) Don't know

8. Which one of the following statements is correct regarding the time that children spend watching electronic products?
□ (1) No more than 1 h per day, 30 min at most each time, and at least 5 min rest
□ (2) No more than 1 h per day, 30 min at most each time, and rest at least 10 min
□ (3) No more than 2 h a day, 40 min at most each time, and rest at least 10 min
□ (4) No more than 2 h a day, 40 min at most each time, and rest at least 10 min
□ (5) Don't know

9. When children use electronic products, how far should they keep their eyes from the screen?
□ (1) The distance between the eyes and the mobile phone is maintained at 30 cm
□ (2) The distance between the eyes and the tablet is maintained at 30 cm
□ (3) The distance between the eyes and the TV, the closer the better under the premise of seeing clearly
□ (4) The distance between the eyes and the TV, as far as possible under the premise of seeing clearly, the better
□ (5) Don't know

10. Regarding outdoor activities, which of the following is wrong?
□ (1) Going outdoors when the sun is strong at noon has the best effect on vision care
□ (2) Outdoor activities for at least 2 h a day are an effective way to prevent myopia
□ (3) Appropriate natural light outdoors can stimulate the release of dopamine, thereby inhibiting the growth of the eye axis and preventing myopia
□ (4)The outdoor activities here refer to any activities or sports performed outdoors during the day.
□ (5) Don't know

Question sets D2 (16 items): Parents' Belief in Vision Care for Children's Use of Electronic Products
Answer instructions: We wish to know your true feelings about your child's vision care using electronic products (TV, computer, mobile phone or tablet). Please check the most suitable answer for each question.

Your true feelings about the vision care of your child using electronic products	Strongly Disagree (1)	Disagree (2)	Undecided (3)	Agree (4)	Strongly Agree (5)
1. I think that children who often watch electronic products can easily lead to premature myopia (eye strain)					
2. I think that children who often watch electronic products can easily lead to deepening of myopia					

3. I think that children who often look at electronic products can easily cause other eye diseases (such as dry eyes, itching)	
4. I think it's a serious matter that my child has myopia prematurely due to frequent viewing of electronic products	
5. I think it's a serious matter that the child's myopia is deepened due to frequent viewing of electronic products.	
6. I think that other eye diseases (such as dry eyes and itching) caused by children who often look at electronic products are serious.	
7. I think that when a child watches electronic product, remind him (her) that the total time per day does not exceed 1 h, which will help his (her) vision care	
8. I think that when a child looks at electronic products, remind him (her) to rest for at least 10 min at most 30 min at a time, which will help his (her) vision care	
9. I think that a child's outdoor activities for at least 2 h a day will help his or her vision care	
10. I think that taking the child to the ophthalmologist to check the eyesight every six months will help his or her eyesight care	
11. Facing the child's request, I find it difficult to restrict him or her from viewing electronic products	
12. I find it difficult to ask children to watch electronic	

products for no more than 1 h a day	
13. I think it's very difficult for a child to take a break of at least 10 min at most 30 min at a time when watching electronic products	
14. I find it difficult to take my children to outdoor activities for at least 2 h a day	
15. . I think it is expensive to take vision care for children (such as purchasing suitable tables and chairs or lighting equipment, vision check, etc.)	
16. I find it inconvenient to take my child to the ophthalmologist for a vision check	

Question sets D3 (4 items): Parents' Action Clues Scale for Children's Vision Care in Using Electronic Products
Instructions for answering: We wish to know under which of the following situations you have received information about vision care. Please read the questions carefully and select the most suitable answer for each question.

Under which of the following situations have you obtained information about vision care for using electronic products?	Yes	No
1. I have participated in education courses related to "Eye Care Using Electronic Products"		
2. I have received information about "eye care using electronic products"		
3. The kindergarten teacher once told me how to take care of my children's vision		
4. The ophthalmologist once told me how to take care of my child's vision		

Question sets D4 (11 items): Parents' Effective Efficacy for Children's Vision Care and Management of Electronic Products
Answering instructions: We wish to know your confidence in your child's use of electronic products in the vision care management. Please read the questions carefully, and select the most suitable answer for each question.

Your confidence ratio in the following situations is	0	25%	50%	75%	100%
1. When a child uses electronic products too close, I can remind him to keep his eyes away from the screen					
2. When the child looks at electronic products, I can remind him to keep his posture upright					
3. When the child looks at electronic products, I can remind him to rest for at least 10 min at most 30 min at a time					

4. When a child looks at electronic products, no matter what he asks, I can remind him that the total time per day cannot exceed 1 h	
5. When the child looks at electronic products, I can remind him to have enough light or lighting in the environment	
6. No matter what the child asks, I can restrict him or her from watching electronic products before going to bed	
7. No matter what the child asks, I can restrict him or her from eating and watching electronic products	
8. No matter what the child asks, I can restrict him or her from watching electronic products on the moving vehicles	
9. Even if it is busy on weekends or holidays, I can let my children have at least 2 h of outdoor activities every day	
10. Even if it is inconvenient, I can still take my child to the ophthalmologist to check the eyesight every six months (not including the kindergarten's eyesight check for young children)	
11. I can persuade my family and my child to agree on the rules for using electronic products	

Question sets D5 (11 items): Parents' Behavior Scale for Vision Care and Discipline of Young Children's Use of Electronic Products
Instructions for answering: This section wants to learn about your child's use of electronic products in the past week. Please read the questions carefully and select the most suitable answer.

In the past week, have you had any of the following situations	Never	Rarely	Sometimes	Often	Always
1. I can remind my child to keep a proper distance when he/she uses the screen					
2. When the child looks at electronic products, I remind him/her to keep his posture upright					
3. When the child looks at electronic products, I remind him/her to rest for at least 10 min at most 30 min at a time					
4. When the child looks at electronic products, I remind him/her that the total time per day cannot exceed 1 h					
5. When the child looks at electronic products, I remind him/her to have enough light or lighting in the environment					
6. I restrict my child not to watch electronic products before going to bed					
7. I restrict children from eating and watching electronic products					
8. I restrict my children not to watch electronic products on moving vehicles					
9. On weekends or holidays, I let my children have at least 2 h of outdoor activities every day					
10. After the kindergarten school on weekdays, I let the children have at least 1 h of outdoor activities every day					

11. I persuaded my family and my child to agree on the rules for using electronic products	

This concludes all the questions, thank you very much for your assistance! Good health!
